# Regadenoson cardiovascular magnetic resonance myocardial perfusion imaging predicts need for future revascularization

**DOI:** 10.1186/1532-429X-14-S1-P7

**Published:** 2012-02-01

**Authors:** Benjamin H Freed, Kristen M Turner, Chattanong Yodwut, Giacomo Tarroni, Emily Estep, Nicole M Bhave, Akhil Narang, Sara Tanaka, Cristiana Corsi, Etienne Gayat, Peter Czobor, Kevin Cavanaugh, Roberto Lang, Victor Mor-Avi, Amit R Patel

**Affiliations:** 1 Internal Medicine, University of Chicago Medical Center, Chicago, IL, USA; 2 Internal Medicine, Loyola Medical Center, Maywood, IL, USA; 3 Internal Medicine, Ramathibodi Hospital, Mahidol University, Bangkok, Thailand; 4 University of Bologna, Bologna, Italy, USA

## Summary

Regadenoson is a new vasodilator myocardial stress agent that is easier-to-use and more tolerable than adenosine. We demonstrate that, in patients undergoing cardiovascular magnetic resonance myocardial perfusion imaging, regadenoson is safe and effective in producing hyperemia and identifying the need for future revascularization.

## Background

Regadenoson (Lexiscan; Astellas) is a new vasodilator myocardial stress agent that selectively activates the A2A receptor. Unlike adenosine, regadenoson is easier to administer and results in fewer side effects. Although extensively studied in patients undergoing nuclear myocardial perfusion imaging (MPI), its performance in cardiovascular magnetic resonance (CMR) MPI remains unknown. The aim of this study was to assess the safety and tolerability of regadenoson and determine its ability to produce hyperemia and predict subsequent coronary revascularization in patients undergoing CMR-MPI.

## Methods

120 patients were prospectively enrolled to receive CMR-MPI (Achieva, Philips 1.5T) with regadenoson. Patients with contraindications to CMR-MPI or regadenoson were excluded. Short-axis slices were obtained at three levels of the left ventricle (LV) during first pass of Gadolinium-DTPA(0.075 mmol/kg at 4 ml/sec) for 50 consecutive heart beats. Images were acquired using a hybrid gradient echo/echo planar imaging sequence. Imaging was performed 1 minute after injection of regadenoson (0.4mg) and then repeated 15 minutes after injection of aminophylline (125mg) under resting conditions. Perfusion defects were defined as subendocardial hypointensity in a coronary distribution at stress, involving ≥25% wall thickness, and persisting for ≥2 heart beats following peak enhancement of the LV cavity. In a subgroup of patients (n=99), custom software was used to generate time intensity curves and to compare the myocardial upslope of the midventricular slice during stress and rest. All subjects were followed for 3 months for the occurrence of coronary revascularization.

## Results

**Figure 1:**
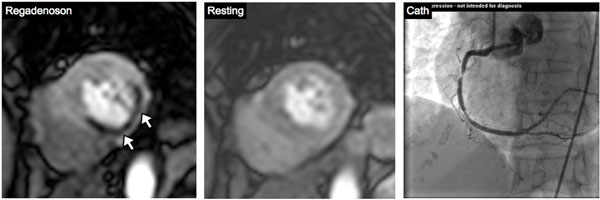
Patient with perfusion defect in inferior and inferior-lateral myocardial wall undergoing regadenoson CMR-MPI and subsequent cardiac catheterization of right coronary artery.

**Figure 2:**
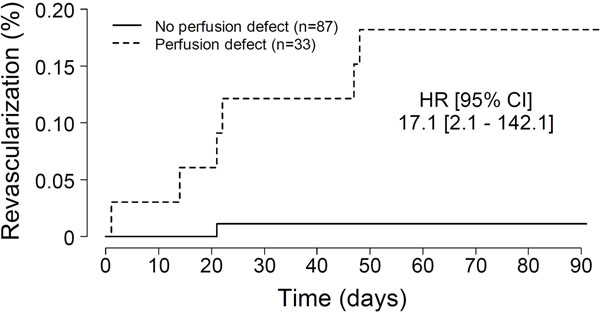
Cumulative incidence of coronary revascularization in patients with and without perfusion defects undergoing regadenoson CMR-MPI.

Overall, 51/120 (43%) of patients were female with an average age of 55±15 years and body mass index of 29±6 kg/m2. Baseline patient characteristics include: coronary artery disease (33%), diabetes (38%), hypertension (56%), and hypercholesterolemia (95%). The average resting blood pressure and heart rate were 124/61mmHg and 70bpm, respectively. Peak heart rate after regadenoson administration was 98bpm (p<0.001). Most patients (87%) experienced side effects from regadenoson including shortness of breath (34%), flushing (23%), and chest discomfort (17%). No EKG changes or residual side effects persisted in any patient at completion of study. The average myocardial upslope increased significantly between rest and stress conditions (9.1±5.9 vs. 12.8±8.1, p<0.001), reflecting the expected hyperemic effect of regadenoson. Perfusion defects were visually apparent in 33/120 (28%) patients. Revascularization occurred in 8/120 (7%) patients (Figure 1). The presence of a perfusion defect significantly predicted future revascularization (Figure 2). Only 1 patient without a perfusion defect was revascularized within the follow-up period.

## Conclusions

Regadenoson is safe, easy-to-use, and effective in producing hyperemia and identifying need for future revascularization in patients undergoing CMR-MPI.

## Funding

Research was funded in part by a research grant from Astellas Pharmaceutical.

